# Supplementary material for: Gut microbiota mediate the FGF21 adaptive stress response to chronic dietary protein-restriction in mice
Source: Nat Commun. 2021 Jun 22;12:3838. doi: 10.1038/s41467-021-24074-z (PMC8219803; doi:10.1038/s41467-021-24074-z)
Supplement: Supplementary file 1 — Supplementary Information [file 41467_2021_24074_MOESM1_ESM.pdf]

Supplementary Fig. 1

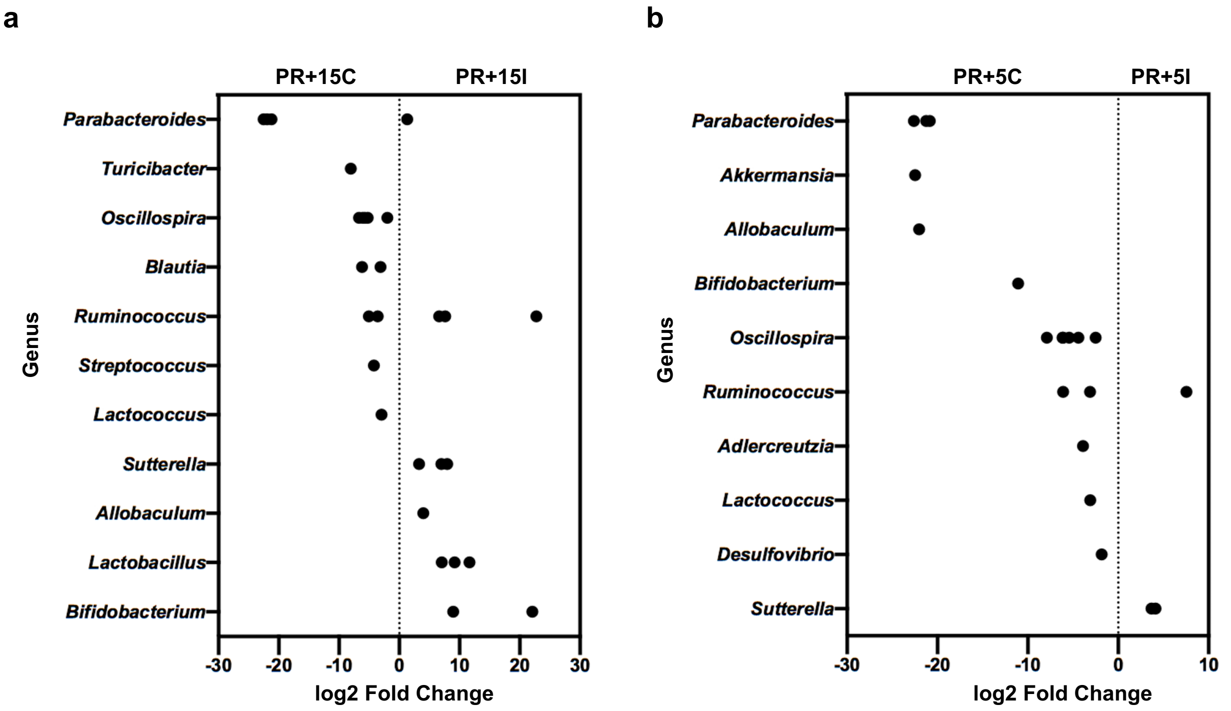

Supplementary Fig. 1: a-b Genus level comparison of significant and differentially abundant bacterial genera using DESeq2 at week 3 in fecal samples from PR+15C vs. PR+15I and PR+5C vs. PR+5I, respectively. All data is represented by n=5 biologically independent animals. PS = protein-sufficient, PR = protein-restricted, C = cellulose, I = inulin.

Supplementary Fig. 2

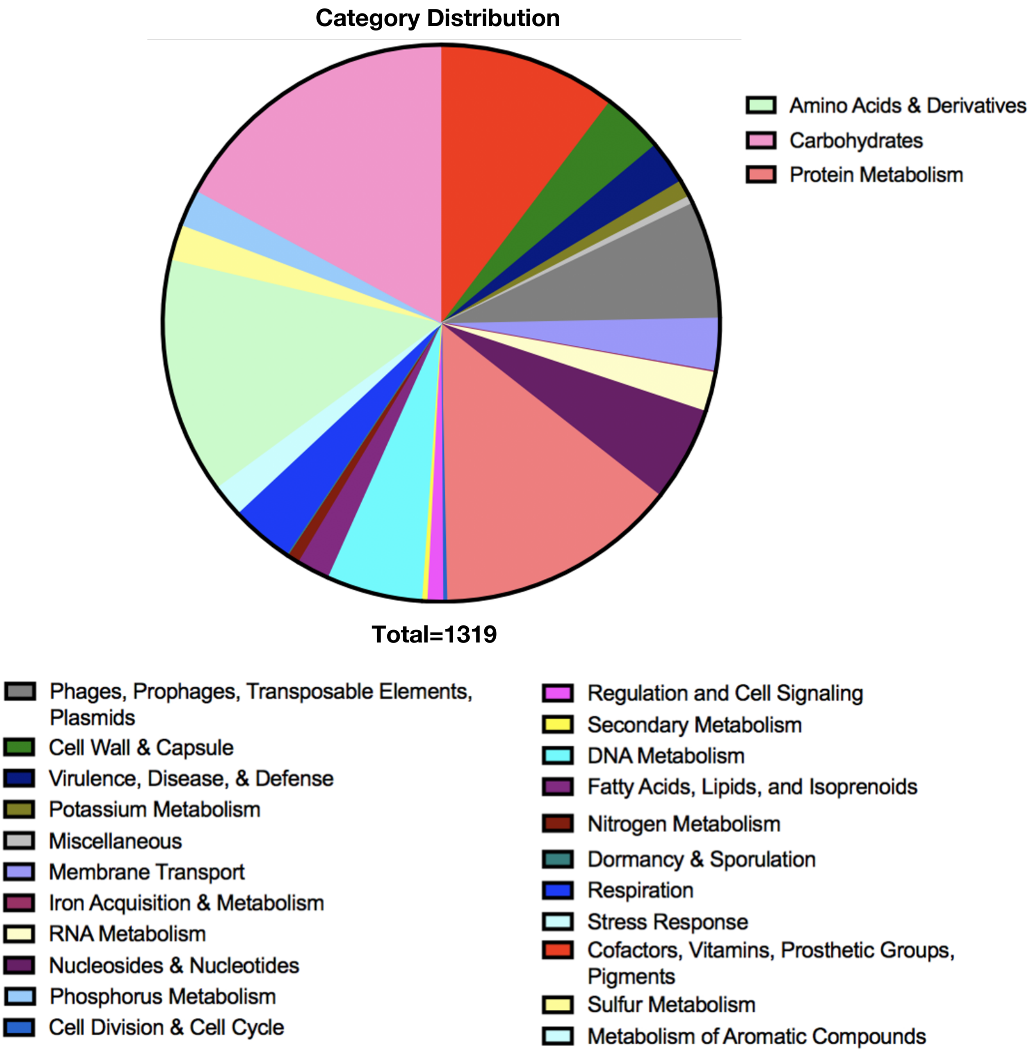

Supplementary Fig. 2: Whole genome sequencing analysis of murine-derived *P. distasonis* displaying functional category distribution.

Supplementary Table 1

Amino Acid Biosynthesis

Subsystem	Role
Histidine Biosynthesis	Phosphoribosylformimino-5-aminoimidazole carboxamide ribotide isomerase (EC 5.3.1.16)
	Histidinol-phosphate aminotransferase (EC 2.6.1.9)
	Phosphoribosyl-ATP pyrophosphatase (EC 3.6.1.31)
	Imidazoleglycerol-phosphate dehydratase (EC 4.2.1.19)
	Phosphoribosyl-AMP cyclohydrolase (EC 3.5.4.19)
	Histidinol-phosphatase (EC 3.1.3.15)
	Histidinol dehydrogenase (EC 1.1.1.23)
Methionine Biosynthesis	Homoserine dehydrogenase (EC 1.1.1.3)
	S-adenosylmethionine synthetase (EC 2.5.1.6)
	O-succinylhomoserine sulfhydrylase (EC 2.5.1.48)
	Serine acetyltransferase (EC 2.3.1.30)
	Homoserine O-succinyltransferase (EC 2.3.1.46)
	5-methyltetrahydrofolate--homocysteine methyltransferase (EC 2.1.1.13)
	Cysteine synthase (EC 2.5.1.47)
	O-acetylhomoserine sulfhydrylase (EC 2.5.1.49)
	Cystathionine beta-lyase (EC 4.4.1.8)
	5,10-methylenetetrahydrofolate reductase (EC 1.5.1.20)
	Methionine transporter MetT
	Adenosylhomocysteinase (EC 3.3.1.1)
Threonine & Homoserine Biosynthesis	Homoserine dehydrogenase (EC 1.1.1.3)
	Aspartate-semialdehyde dehydrogenase (EC 1.2.1.11)
	Aspartate aminotransferase (EC 2.6.1.1)
	Threonine synthase (EC 4.2.3.1)
	Aspartokinase (EC 2.7.2.4)
	Predicted functional analog of homoserine kinase (EC 2.7.1.-)
Threonine Degradation	Threonine dehydrogenase and related Zn-dependent dehydrogenases
	low-specificity D-threonine aldolase
	Threonine dehydratase, catabolic (EC 4.3.1.19)
	L-threonine 3-dehydrogenase (EC 1.1.1.103)
Lysine Biosynthesis DAP Pathway	Diaminopimelate decarboxylase (EC 4.1.1.20)
	L,L-diaminopimelate aminotransferase (EC 2.6.1.83)
	Diaminopimelate epimerase (EC 5.1.1.7)
	Aspartate-semialdehyde dehydrogenase (EC 1.2.1.11)
	4-hydroxy-tetrahydrodipicolinate reductase (EC 1.17.1.8)
	Aspartokinase (EC 2.7.2.4)
	4-hydroxy-tetrahydrodipicolinate synthase (EC 4.3.3.7)
	Meso-diaminopimelate D-dehydrogenase (EC 1.4.1.16)

Cysteine Biosynthesis	Sulfate adenylyltransferase subunit 2 (EC 2.7.7.4)
	Sulfate adenylyltransferase subunit 1 (EC 2.7.7.4)
	Cysteine synthase (EC 2.5.1.47)
	Sulfate permease
	Adenylylsulfate kinase (EC 2.7.1.25)
	Serine acetyltransferase (EC 2.3.1.30)

Supplementary Table 1: KEGG enzymes in *P. distasonis* amino acid biosynthetic pathways

Supplementary Table 2

Ingredient (g)	PS (18%)	PS (18%) + 5% Cellulose	PS (18%) + 5% Inulin	PR (8%)	PR (10%)	PR (10%) + 5% Cellulose	PR (10%) + 5% Inulin	PR (10%) + 15% Cellulose	PR (10%) + 15% Inulin
Casein	200.0	200.0	200.0	87.0	115.0	115.0	115.0	115.0	115.0
L-cystine	3.0	3.0	3.0	1.3	1.7	1.7	1.7	1.7	1.7
Corn Starch	462.1	412.2	412.1	565.3	536.6	486.7	486.7	404.7	404.7
Maltodextrin	132.0	132.0	132.0	150.0	150.0	150.0	150.0	132.0	132.0
Sucrose	100.0	100.0	100.0	100.0	100.0	100.0	100.0	100.0	100.0
Soybean Oil	50.0	50.0	50.0	50.0	50.0	50.0	50.0	50.0	50.0
Cellulose	0.0	50.0	0.0	0.0	0.0	50.0	0.0	150.0	0.0
Inulin	0.0	0.0	50.0	0.0	0.0	0.0	50.0	0.0	150.0
Mineral Mix (98057)	0.0	0.0	0.0	13.4	13.4	13.4	13.4	13.4	13.4
Mineral Mix (94046)	35.0	35.0	35.0	0.0	0.0	0.0	0.0	0.0	0.0
Calcium Phosphate, dibasic	0.0	0.0	0.0	10.5	12.2	12.2	12.2	12.2	12.2
Calcium Carbonate	0.0	0.0	0.0	4.7	3.3	3.3	3.3	3.3	3.3
Vitamin Mix, AIN-93-VX (94047)	15.0	15.0	15.0	15.0	15.0	15.0	15.0	15.0	15.0
Choline Bitartrate	2.8	2.8	2.8	2.8	2.8	2.8	2.8	2.8	2.8
Vitamin K1, phylloquinone	0.002	0.002	0.002	0.002	0.002	0.002	0.002	0.002	0.002
TBHQ, antioxidant	0.01	0.01	0.01	0.01	0.01	0.01	0.01	0.01	0.01
Kcal/g	3.8	3.7	3.7	3.8	3.8	3.6	3.7	3.3	3.5
Protein (% by weight)	18.0	18.0	18.0	8.0	10.0	10.0	10.0	10.0	10.0
Carbohydrate (% by weight)	66.0	62.0	63.0	77.0	74.0	70.0	71.0	60.0	65.0
Fat (% by weight)	5.0	5.0	5.0	5.0	5.0	5.0	5.0	5.0	5.0

Supplementary Table 2: Diet formulations. PS = protein-sufficient, PR = protein-restricted.

Supplementary Table 3

Gene	Forward	Reverse
Asns	5’-TACAACCACAAGGCGCTACA-3’	5’-AAGGGCCTGACTCCATAGGT-3’
Fgf21	5’-TAATAGTGCCCTGGACG-3’	5’-GCAGAGTCACCATTGTAGTA-3’
Rps17	5’-GAATGCTGTTTGATACTGTG-3’	5’-TTTTAAGGAAAAACATACAGG-3’
3pgd	5’-GAGAAAATCCGAAAGAAATA-3’	5’-TGACCAACTGTAAGATCAGA-3’
27F / 1492R	5’-AGAGTTTGATCMTGGCTCAG-3’	5’-TACGGYTACCTTGTTACGACTT-3’

Supplementary Table 3: Primers used for PCR
