# Supplementary material for: Gut microbiota mediate the FGF21 adaptive stress response to chronic dietary protein-restriction in mice
Source: Nat Commun. 2021 Jun 22;12:3838. doi: 10.1038/s41467-021-24074-z (PMC8219803; doi:10.1038/s41467-021-24074-z)
Supplement: Supplementary file 3 — Reporting Summary New [file 41467_2021_24074_MOESM3_ESM.pdf]

nature research

Corresponding author(s): Suzanne Devkota

Last updated by author(s): May 14, 2021



# Reporting Summary

Nature Research wishes to improve the reproducibility of the work that we publish. This form provides structure for consistency and transparency in reporting. For further information on Nature Research policies, see our Editorial Policies and the Editorial Policy Checklist.

## Statistics

For all statistical analyses, confirm that the following items are present in the figure legend, table legend, main text, or Methods section.

| n/a | Confirmed | |
|---|---|---|
| ☐ | ☒ | The exact sample size (*n*) for each experimental group/condition, given as a discrete number and unit of measurement |
| ☐ | ☒ | A statement on whether measurements were taken from distinct samples or whether the same sample was measured repeatedly |
| ☐ | ☒ | The statistical test(s) used AND whether they are one- or two-sided<br>*Only common tests should be described solely by name; describe more complex techniques in the Methods section.* |
| ☐ | ☒ | A description of all covariates tested |
| ☐ | ☒ | A description of any assumptions or corrections, such as tests of normality and adjustment for multiple comparisons |
| ☐ | ☒ | A full description of the statistical parameters including central tendency (e.g. means) or other basic estimates (e.g. regression coefficient) AND variation (e.g. standard deviation) or associated estimates of uncertainty (e.g. confidence intervals) |
| ☐ | ☒ | For null hypothesis testing, the test statistic (e.g. *F*, *t*, *r*) with confidence intervals, effect sizes, degrees of freedom and *P* value noted<br>*Give P values as exact values whenever suitable.* |
| ☒ | ☐ | For Bayesian analysis, information on the choice of priors and Markov chain Monte Carlo settings |
| ☐ | ☒ | For hierarchical and complex designs, identification of the appropriate level for tests and full reporting of outcomes |
| ☐ | ☒ | Estimates of effect sizes (e.g. Cohen's *d*, Pearson's *r*), indicating how they were calculated |

*Our web collection on statistics for biologists contains articles on many of the points above.*

## Software and code

Policy information about availability of computer code

| | |
|---|---|
| Data collection | No software was used for data collection. |
| Data analysis | Microbiome sequences were subsampled to 20,000 reads per sample prior to diversity assessments. α-diversity (i.e. bacterial variety and complexity within a sample) and β-diversity (differences in composition across diets) were calculated using the phyloseq package v1.30 in R version 3.6.2. Community difference was assessed using Bray-Curtis distance and visualized by principal coordinate analysis. DESeq2 v1.26 was used to test for significant differentially abundant sequence variants between diets. Reads were quality filtered, trimmed, merged, denoised, chimera filtered, and binned into sequence variants using DADA2 v1.14.1. Sequence variants were aligned to the GreenGenes reference database v13.8 ( https://greengenes.secondgenome.com). R version 3.6.2.<br><br>DNA extracts from bacterial isolates was subject to PCR using full length 16s rRNA primers (27F and 1492R). Amplicons were submitted to Laragen for Sanger sequencing, and the resultant sequence traces were visualized in FinchTV v1.4. The trimmed reads were identified by Microbial BLAST (https://blast.ncbi.nlm.nih.gov/Blast.cgi?PAGE_TYPE=BlastSearch&BLAST_SPEC=MicrobialGenomes).<br><br>The raw paired-end reads were quality trimmed using Nesoni pipeline ( https://github.com/Victorian-Bioinformatics-Consortium/nesoni). The quality filtered reads were assembled into a 5.53 Mb genome with 53 contigs and 45.2% G+C content using SPAdes assembler (https://www.ncbi.nlm.nih.gov/pmc/articles/PMC3342519/) at a k-mer length set between 41 and 61. The contigs were then annotated into broad pathway level (i.e. subsystems) and enzyme level features using RAST annotation server for functional roles (https://rast.nmpdr.org). No custom code used.<br><br>The relationship between plasma FGF21 and change in community composition was assessed with Pearson's correlation test in GraphPad Prism v.8.2.1. |

For manuscripts utilizing custom algorithms or software that are central to the research but not yet described in published literature, software must be made available to editors and reviewers. We strongly encourage code deposition in a community repository (e.g. GitHub). See the Nature Research guidelines for submitting code & software for further information.

## Data

Policy information about availability of data

All manuscripts must include a data availability statement. This statement should provide the following information, where applicable:

- Accession codes, unique identifiers, or web links for publicly available datasets
- A list of figures that have associated raw data
- A description of any restrictions on data availability

Source data is now included with the manuscript, and all genomic data has been deposited in NCBI with accession numbers listed under "Data Availability". Specifically, the 16S rRNA and bacterial whole genome sequencing data has been made publicly available through the NCBI SRA database with accession numbers PRJNA726200 [https://www.ncbi.nlm.nih.gov/bioproject/PRJNA726200/] and PRJNA726208 [https://www.ncbi.nlm.nih.gov/sra/PRJNA726208], respectively.

Databases used w/ links:
The raw paired-end reads were quality trimmed using Nesoni pipeline (available at- https://github.com/Victorian-Bioinformatics-Consortium/nesoni).
The quality filtered reads were assembled into a 5.53 Mb genome with 53 contigs and 45.2% G+C content using SPAdes assembler (https://www.ncbi.nlm.nih.gov/pmc/articles/PMC3342519/) at a k-mer length set between 41 and 61.
The contigs were then annotated into broad pathway level (i.e. subsystems) and enzyme level features using RAST annotation server for functional roles (https://bmcgenomics.biomedcentral.com/articles/10.1186/1471-2164-9-75).

# Field-specific reporting

Please select the one below that is the best fit for your research. If you are not sure, read the appropriate sections before making your selection.

☒ Life sciences ☐ Behavioural & social sciences ☐ Ecological, evolutionary & environmental sciences

For a reference copy of the document with all sections, see nature.com/documents/nr-reporting-summary-flat.pdf

# Life sciences study design

All studies must disclose on these points even when the disclosure is negative.

| | |
|---|---|
| Sample size | A sample size of n=10 mice per diet group was determined based on 12 years of performing diet and microbiome studies in mice. We have determined a minimum of 8 mice per group is required to achieve statistical significance across our diet studies in C57Bl/6 mice, and is consistent with other published diet and microbiome studies in mice (Devkota S., et al., Nature 487, 104–108, 2012; Upadhyay V, et al. Nat Immunol. 13(10):947-953, 2012; Martinez-Guryn et al., Cell Host & Microbe, 23(4):458-469, 2018.) |
| Data exclusions | No data has been excluded. |
| Replication | We have verified reproducibility of our data by conducting our animal experiments over a staggered time period where multiple litters of mice had to be used. Despite performing and repeating several of these analysis over different time periods (original submission and revised submission) the resulting data has been consistent. Furthermore, some aspects of our data were carried out to establish we could reproduce pre-existing findings in the literature, and indeed, our results are consistent with other studies that have used similar treatments. For host measurments, n=10 biologically independent animals or as indicated in figure legends. For 16s rRNA sequencing analysis, a subset of n=5 per group of biologically independent animals are analyzed. |
| Randomization | All mice used in these experiments were of the same age- the only variables were starting body weight and litter. All candidate mice were first weighed and outliers were removed. Then only mice within +/- 1 gram of the remaining mean were used. Within this, mice from the same litters were spread across diet treatments so as not to create a littermate bias. Thus each diet group consisted of age-matched and weight-matched mice, with at least 2 litters represented. |
| Blinding | Our Research Technician administered the diets and weighing the mice were blinded to the diet treatment groups as each diet was colored with a different food coloring. Therefore he only presented the diets based on color not content to the mice. However, my post-doc who designed the diets also performed the data analysis and thus could not be blinded to the samples. |

# Reporting for specific materials, systems and methods

We require information from authors about some types of materials, experimental systems and methods used in many studies. Here, indicate whether each material, system or method listed is relevant to your study. If you are not sure if a list item applies to your research, read the appropriate section before selecting a response.

## Materials & experimental systems

| n/a | Involved in the study | |
|---|---|---|
| ☐ | ☒ | Antibodies |
| ☒ | ☐ | Eukaryotic cell lines |
| ☒ | ☐ | Palaeontology and archaeology |
| ☐ | ☒ | Animals and other organisms |
| ☒ | ☐ | Human research participants |
| ☒ | ☐ | Clinical data |
| ☒ | ☐ | Dual use research of concern |

## Methods

| n/a | Involved in the study | |
|---|---|---|
| ☒ | ☐ | ChIP-seq |
| ☒ | ☐ | Flow cytometry |
| ☒ | ☐ | MRI-based neuroimaging |

# Antibodies

| | |
|---|---|
| Antibodies used | We only used antibodies for the western blots and these are listed in the Methods section under Western Blot. Antibody manufacturer, lot number, and dilutions are described in Methods and below:<br>Anti-eIF2 (Abcam, Cambridge, MA; Cat # ab169528, Lot # GR321237-4; 1:1000 dilution), Anti-phospho-eIF2 (Cell Signaling, Danvers, MA; Cat # 3398S, Lot # 6, 1:500 dilution), and Anti-β-Tubulin (Sigma Aldrich, St. Louis, MO, Cat # T4026, Lot # 125M4884V; 1:200 dilution). |
| Validation | We list our antibody dilutions for the western blots in the methods section and the data presented using these antibody's at the described dilutions represent our validation. Primary antibody validation was determined as follows: Anti-eIF2a (Li D  et al. Cell Death Dis 10:744 (2019), and Fig. 3B); Anti-phospho eIF2a (Xu et al.  Int J Mol Med 46(3):1107- 1117 (2020), and Fig. 3B); Anti-β-Tubulin (Bian et al. Nat Commun 8:14420 (2017) and Fig. 3B). |

# Animals and other organisms

Policy information about studies involving animals; ARRIVE guidelines recommended for reporting animal research

| | |
|---|---|
| Laboratory animals | These details are reported in the Methods under Animals & Diets and here: C57BL/6 mice raised under specific-pathogen free conditions were housed at Cedars-Sinai Medical Center, Los Angeles, CA, USA and bred for seven generations prior to use to ensure a microbiome adapted to our housing conditions. Male mice at 6-8 weeks of age were used for diet interventions. The mice were housed in a 12-h light:dark cycle at 22°C–25°C temperature with relative humidity of 50–70 percent. For germ-free mouse experiments, eight-week old male C57Bl/6 germ-free (n=5) C57Bl were obtained from Jackson laboratory. The mice were housed in the germ-free facility at the University of California, San Diego, CA, USA and allowed to acclimate for 14 days. These mice were housed in a 12-h light:dark cycle at 20°C -22°C temperature with relative humidity of 30-79 percent. |
| Wild animals | No wild animals were used in this study. |
| Field-collected samples | There are no field-collected samples in this study. |
| Ethics oversight | This is listed in the Methods section under Animals & Diets |

Note that full information on the approval of the study protocol must also be provided in the manuscript.
